# Hepatitis C, from screening to treatment, a revolution

**DOI:** 10.7448/IAS.17.4.19499

**Published:** 2014-11-02

**Authors:** Karine Lacombe

**Affiliations:** Infectious and Tropical Diseases Department, Saint-Antoine Hospital, Paris, France

## Abstract

Initially associated with blood transfusions and hospital care, the hepatitis C virus (HCV) epidemic has since moved rapidly onwards to intravenous drug users, where it poses a major problem still today. Despite numerous harm reduction programs, such as needle exchange, opiate substitution and controlled shooting galleries, drug addiction remains the number one route of HCV transmission around the world. However, in HIV-positive patients, the HCV epidemic experienced a major shift in the mid-90s, with sexual transmission among particularly men who have sex with men (MSM) surfacing as a major route of HCV acquisition. Until recently, therapeutic options in HCV infection had been quite limited, based largely on the use of combined Peg-interferon and ribavirin, with disappointing results in HIV-positive patients. With the recent, exciting developments in HCV research, new targets on the virus replication cycle have been discovered. After the release in 2011 of the first direct antiviral agents inhibiting the NS3/4 antiprotease, the development of drugs with newer mode of action, more efficient and with higher tolerance profiles has greatly accelerated. They may greatly change the course of treatment: once daily, highly active and easily-tolerated regimens, administered for a short period of time with HCV eradication in more than 90% of patients, independently of prior treatment, level of fibrosis and comorbidities such as HIV co-infection. This definitely has the potential to curb the HCV epidemic towards an HCV cure, provided that advocacy for broad-access to treatment is successful for those most in need. Their use in resource-constrained settings may also be the next political challenge for HCV.

